# Trace Mineral Nutrition of Grazing Beef Cattle

**DOI:** 10.3390/ani11102767

**Published:** 2021-09-22

**Authors:** John D. Arthington, Juliana Ranches

**Affiliations:** 1 Department of Animal Sciences, University of Florida, Gainesville, FL 32611, USA; 2 Eastern Oregon Agricultural Research Center, Oregon State University, Burns, OR 97720, USA; juliana.ranches@oregonstate.edu

**Keywords:** beef, cows, grazing, trace minerals

## Abstract

**Simple Summary:**

The trace mineral nutrition of grazing beef cattle is an essential, but often complicated component of the management program. Throughout the annual cycle, forage is the primary source of trace mineral supply to grazing cattle, but concentrations vary depending on a multitude of factors. Trace mineral deficiencies are common when relying solely on forage to meet cattle requirements. Selenium, Cu, Zn, Mn, Co, and I are the trace minerals most commonly found to be deficient in forage. Trace mineral antagonists, such as Fe, Mo, and S, are commonly found in forage and exist in varying concentrations further complicating the success of satisfying the requirement of grazing cattle. Trace mineral-fortified, salt-based, free-choice supplements are the most common supplementation strategies available. Cattle voluntarily consume these supplements to satisfy their salt craving and thus indirectly receive supplemental trace minerals. Managing salt inclusion and seasonal variation in voluntary intake are essential to the success of this management system. Supplements can be formulated with a variety of trace mineral ingredients available to the industry, which are generally grouped into different source categories. Other supplementation strategies to supply trace minerals to grazing cattle include fortification of energy and protein supplements, biofortification, injectable trace minerals, and boluses.

**Abstract:**

The trace mineral requirements of grazing beef cattle are often complicated by different environmental factors, such as the lack of specific trace minerals or the presence of trace mineral antagonists in forage. Nearly every region of the world has specific implications related to trace mineral nutrition of grazing cattle. Since forage is the most significant contributor to trace mineral nutrition, it is important to consider the concentrations of trace minerals and antagonists and how they may impact the performance of cattle consuming them. This review attempts to provide an update on the trace minerals commonly found to be inadequate in forage, supplementation strategies to address deficiency including a discussion on supplemental trace mineral source, and the complications presented by mineral antagonists. Although the review focuses on beef cattle grazing systems of the United States, the information herein is derived from both extensive native range and intensive planted pasture.

## 1. Introduction

The trace mineral requirements for optimal health and performance of beef cattle are continually becoming better understood. As a result, research findings have implicated only minimal changes in suggested requirements over the last several decades. For grazing beef cattle, trace mineral adequacy is largely based on the trace mineral content of the forage being consumed, which is impacted by forage specie, maturity, fertilization, and soil pH and mineralization. With the typical exception of Na, some forages at certain times of the year may fully supply the trace mineral requirement of the animal. Even during periods of forage trace mineral inadequacy, the animal may have adequate body stores to call upon to protect against complications associated with short-term deficiency. In addition, most grazing cattle are provided with supplemental nutrients to address forage deficiencies in protein and energy. These management practices also add to the total trace mineral supply consumed by the animal. To complicate the issue further, trace mineral adequacy can be compromised by the presence of antagonists in forages and supplements. Each of these factors contribute to the complexity of managing the optimal trace mineral status of grazing cattle. Nearly every region of the world has specific trace mineral implications that change the narrative and impact the resulting management system. This is precisely why this area of animal nutrition is both frustratingly difficult and interesting from an animal production perspective. Although this review focuses only on trace mineral nutrition of grazing cattle, earlier reviews provide additional useful information on this topic [1,2,3,4,5]. This review attempts to provide an update on the trace minerals commonly found to be inadequate in forage, supplementation strategies to address deficiency, including a discussion on supplemental trace mineral source, and the complications presented by mineral antagonists. Although the review focuses on beef cattle grazing systems of the United States, the information herein is derived from both extensive native range and intensive planted pasture conditions and has a relevant application to grazing systems throughout the world.

## 2. Essential Trace Minerals

There are 10 trace minerals known to be essential for cattle, including Cr, Co, Cu, I, Fe, Mn, Mo, Ni, Se, and Zn [6]. In terms of essentiality, this list was expanded in the sixth [7] to seventh [8] NRC revision to include Ni and Cr, however without established requirements. The list has remained unchanged in the eighth beef cattle NRC revision [6], which is shown in Table 1. Although other trace minerals may be implicated as essential, their contributions to cattle nutrition remain largely unknown.

Among these 10 essential trace minerals, only 6 hold practical relevance to trace mineral adequacy of grazing cattle. These include Se, Cu, Zn, Mn, I, and Co. Concentrations of these elements in grazed forage range from commonly deficient to generally adequate. A good understanding of local conditions and the management implications impacting trace mineral nutrition in grazed forage are essential.

### 2.1. Selenium

Selenium is known to be the most deficient trace mineral in the diet of cattle consuming forage-based diets. However, this distinction comes with an important dichotomy, in that Se is also uncommonly found to be toxic in plants in specific regions of the world. The range between Se adequacy and toxicity is the least among the 6 commonly supplemented trace minerals. Current recommendations suggest dietary concentrations of 0.1 and 0.3 mg/kg for beef and dairy cattle, respectively. There is no evidence that Se requirements should exceed 0.3 mg/kg of DMI and due to regionalized concerns for Se toxicity, the Food and Drug Administration (FDA) has established upper limits on formulated diets and free-choice supplements (0.3 mg Se/kg and 3 mg Se/d for complete rations and free-choice supplements, respectively).

Selenium is a cofactor in numerous metalloenzyme systems in the body and is most notably known for its role in the antioxidant, glutathione peroxidase. Among the antioxidants, glutathione peroxidase is recognized as one of the most powerful mediators of oxidative stress in mammals. Therefore, it is not surprising that the clinical manifestation of Se deficiency presents as conditions linked to oxidative stress and tissue degeneration, such as White Muscle Disease (WMD). Furthermore, the interactions between Se and vitamin E have been recognized for several years and the most well described and most common clinical manifestation of Se and vitamin E deficiency is the nutritional myodegeneration or WMD [9]. Even in instances where the clinical manifestation of WMD is absent, less severe Se deficiency may be linked to newborn calves experiencing a lack of vigor or thrift, commonly referred to as “Weak Calf Syndrome”.

Other clinical conditions commonly associated with Se deficiency include retained placenta and reductions in immune competence, particularly the function of phagocytic cells. Additionally, Se and S have similar physical and chemical properties allowing high S diets the ability to reduce Se bioavailability resulting in Se deficiency.

Selenium, unlike all other essential trace minerals for cattle, is not essential for higher plants. Its presence in plant tissue is the result of luxury accumulation due simply to its presence in the soil, therefore, accurate soil nutrient mapping is reflective of the Se content of forages grown in specific areas. In the U.S. northern high plains, for example, soil Se concentrations tend to be fairly high [10] and the forages grown in these areas are commonly adequate in Se. This is reflective by the tissue Se concentrations of cattle harvested from these areas [11]. In these regions, additional Se supplementation is typically not required.

### 2.2. Copper

Copper is the second most commonly deficient trace mineral for grazing cattle and certainly in some parts of the world, the prevalence of Cu deficiency exceeds that of Se. Although Cu toxicity with poorly formulated supplementation strategies can be a problem, forage Cu concentrations, different from Se, do not present toxicity concerns. The requirement for Cu in beef cattle was increased from 8 to 10 mg Cu/kg DM from the sixth to seventh NRC revision and remained unchanged in the eighth revision. There is no evidence that grazing cattle require a Cu intake above 10 mg/kg except when antagonists are present. The most common Cu antagonists are S, Mo, and Fe. The influence of S as a Cu antagonist has become more common as the prevalence of S has increased in forages and supplemental concentrates. For forages, this is the result of increased use of ammonium sulfate as a fertilizer N source. This change is a result of increasing difficulty for regional fertilizer suppliers to inventory ammonium nitrate due to elevated public safety concerns [12]. Arthington et al. [13] reported lower Cu liver concentration for cows grazing bahiagrass (*Paspalum notatum*) pastures fertilized with ammonium sulfate when compared to cows grazing pastures fertilized with ammonium nitrate, while having free-choice access to a complete salt-based trace mineral supplement.

Copper is essential for a variety of metalloenzymes, which largely participate in the coordination of normal immune function. Among these Cu-dependent enzymes, ceruloplasmin is the most notable. Ceruloplasmin is associated with nearly 90% of Cu found in blood and is known as a major bovine acute phase protein. During instances of inflammatory distress, ceruloplasmin concentrations increase sharply in Cu-adequate cattle. However, in Cu-deficient cattle, this response is suppressed resulting in an alteration in the normal acute phase reaction, with notable increases in haptoglobin and fibrinogen concentrations [14,15]. The acute phase protein response is a normal physiological response of the innate immune system and is elicited in cattle in response to both disease [15,16] and vaccination [17,18].

A recent advancement in our knowledge of clinical Cu deficiency relates to the direct role of Cu on reproductive function. For decades, Cu adequacy has been linked to the support of normal reproductive function in cattle; however, Phillippo et al. [19] published a study that revealed delayed puberty in Cu-deficient heifers that were supplemented with Mo, but not Fe. Although both antagonists reduced Cu status, only the Mo-supplemented heifers experienced delayed puberty. Heifer average daily gain (ADG) was not a contributing factor, as heifers consuming a control diet had similar body weight (BW) gain as Mo-supplemented heifers, but normal puberty attainment. This study was the first to implicate Mo, independent of Cu, as the primary nutritional factor impacting reproduction in cattle. Studies from the University of Nottingham have advanced this topic further to reveal that thiomolybdates (Mo × S complexes) [20] are responsible for impacting normal ovarian steroidogenesis [21] likely through impacts upon the Cu-dependent enzyme, lysyl oxidase [22]. In the United Kingdom, this condition in cattle is often referred to as “Copper Responsive Infertility”, because it can often be reversed with adequate Cu supplementation. Because of their work, we now understand that it is Mo and S, and not Cu that is impacting cattle infertility. Current reviews of this topic have been published by other authors covering pre-absorption [23] and post-absorption [24] research on the influence of Cu and thiomolybdates in cattle.

Today, most researchers agree that the direct effect of Cu deficiency in cattle is manifested through reductions in immune competence [25]. This likely begins with altered innate immune responses through perturbation of the acute phase response (i.e., ceruloplasmin activity) and continues through reductions in both humoral and phagocytic cell function [15,26,27].

### 2.3. Zinc

Zinc is the third most commonly deficient trace mineral for grazing cattle. The Zn requirement for beef cattle has remained consistent at 30 mg/kg DM through the sixth, seventh, and eighth revisions of the NRC; however, the eighth revision did point out that Zn requirements for grazing cattle are not well defined. Zinc lacks a reliable tissue pool to assess animal status. Whereas whole blood Se and liver Se and Cu concentrations serve as good indicators of overall animal status, neither tissue provides an accurate indicator of Zn status. In addition, Zn also lacks a reliable well-defined enzyme for monitoring status, which is existing for Cu (ceruloplasmin and superoxide dismutase) and Se (glutathione peroxidase). This diagnosis pitfall likely has contributed to our inability to better define and/or diagnose production implications resulting from Zn deficiency. In addition, Zn deficiency in grazing cattle is likely to be rare, as the Zn concentration of most forages tend to be marginally adequate. In a survey of 23 U.S. states, Mortimer et al. [28] found that pasture/range grass Zn concentrations averaged 29 ± 9.2 mg/kg with fairly consistent results across four geographic regions of the U.S. (range = 19.5 to 42.9 mg/kg Zn; *n* = 164 samples).

As with Cu and Se, Zn is also essential for a multitude of metalloenzymes. Most notable is the role of Zn on RNA and DNA metabolism, which explains its commonly known connection to hoof health [29] and other physiological processes involving rapidly dividing cells, such as spermatogenesis [30] and immune function [31]. Furthermore, Zn supplementation of growing cattle has been shown to influence hormone receptor signaling, circulating insulin-like growth factor-1 concentrations, glucose metabolism, protein synthesis via mTOR, and satellite cell proliferation and differentiation, factors that have been discussed comprehensively in a review by Messersmith et al. [32].

### 2.4. Manganese

Manganese is commonly included in supplements provided to grazing beef cattle, but is seldom lacking in the forage. The Mn requirement for beef cattle has remained consistent at 40 mg/kg DM for breeding cattle and 20 mg/kg DM for growing and finishing cattle through the sixth, seventh, and eighth revisions of the NRC. There are very few reported instances of Mn deficiency in grazing cattle; however, there is no reliable biomarker for diagnosis of Mn deficiency. The Mn values in the blood, bones, and liver decline in animals deprived of Mn but such markers are not as reliable as liver for elements such as Cu and Se [33]. In experimental conditions, [34] calves born to heifers maintained on a Mn-deficient diet (approximately 16 mg Mn/kg DM) prior to and during gestation have shown multiple birth defects and less BW at birth when compared to calves born to Mn-supplemented heifers. In younger animals, signs of Mn deficiency include skeletal abnormalities [35,36,37], where calf deformities are observed as superior bachygnathism, dwarfism, and swollen joints, resulting in the lack of ability to stand and walk [37] as observed by Hansen et al. [34]. Additionally, irregular estrous and low conception rates are other reported signals of Mn deficiency in cattle [6]. In a review, Spears [37] highlights the function of three mammalian metalloenzymes that have been identified as Mn-dependent, therefore requiring Mn for normal functioning of the enzyme. The Mn-dependent superoxide dismutase is important in the antioxidant system and is located in the mitochondria; arginase is an Mn-containing enzyme involved in the urea cycle; and the glycosyl transferase is an enzyme activated by Mn, which is required for synthesis of proteoglycans in cartilage tissue.

Although variable, Mn concentrations of forages are commonly at or above the recommended 40 mg/kg of DM. High levels of Mn are tolerated by grazing beef cattle and toxicity is rare, likely due to the low intestinal absorption rate of Mn and great liver capacity to excrete Mn via the bile [37].

### 2.5. Cobalt

Cobalt is essential for the ruminal synthesis of vitamin B12. The eighth revision of the beef cattle NRC increased the Co requirement from 0.10 to 0.15 mg/kg of DM. This was in response to a series of in vitro and in vivo studies involving concentrate feeds offered to growing and finishing cattle. These authors concluded that dietary Co concentrations of 0.15 mg/kg were required for optimal vitamin B12 synthesis in the rumen [38,39]. Although most supplements for grazing beef cattle target Co intakes at or above 0.15 mg/kg of DMI, studies have not implicated improved performance of grazing cattle, particularly breeding cattle, as a result of increased Co intake.

Cobalt deficiency can occur after grazing Co-deficient pastures for long periods of time. Early signs of Co deficiency include lack of appetite accompanied by reduced BW gain. Furthermore, Co deficiency can negatively impact the metabolism of vitamin B12, which in turns disrupts lipid metabolism, through effects on the enzymes, methyl malonyl- CoA mutase and methionine synthase [40]. Prior to understanding the essentiality of Co to grazing cattle, producers recognized the deficiency condition and associated it with certain pastures. Producers would plan to move cattle to “healthy” pastures prior to the onset of clinical symptoms [41]. Cobalt toxicity is rare, and cattle can tolerate as much as 100 times the dietary requirement [42].

### 2.6. Iodine

Iodine participates in body energy metabolism through its role in thyroid hormone production (i.e., thyroxin and triiodothyronine). Iodine requirement has remained unchanged at 0.5 mg/kg DM through the sixth, seventh, and eighth revisions of the NRC. Classic clinical signs of I deficiency include goiter, which is characterized by the enlargement of the thyroid gland. Throughout history, I deficiency has been widespread and prevalent, occurring in every country of the world [41]. In modern production systems, I deficiency is rare. This is primarily due to the I-fortification of salt, which has made a dramatic impact on meeting I requirements of both livestock and humans. The requirement of I can also be influence by the presence of goitrogenic substances in the diet, such as thiocyanates and glycosinolates, which impairs I uptake by the thyroid. For grazing cattle, the provision of I-fortified salt, along with feed ingredient sources of supplemental I, is adequate to protect against deficiency. Iodine toxicity is rare in grazing cattle.

## 3. Methods of Supplementation

### 3.1. Free-Choice, Salt-Based Supplements

The most common method for providing supplemental trace minerals to grazing cattle involves the formulation and blending of supplemental trace minerals with common salt, offered free choice. This system is based on the assumption that cattle have a “nutrition wisdom” which causes them to seek and consume salt at a level that meets or exceeds their requirement for Na. Through this natural behavior, the deliberate and intentional consumption of salt results in the delivery of supplemental trace minerals. Although largely effective in most grazing situations, this management system is not without complications. Most notable and most influential to success is the necessity to manage free-choice intake. This practice requires careful attention to fluctuations of salt craving and a requirement that the manager understands that the craving for salt is the major factor driving variation in intake. For example, in most tropical and subtropical grazing climates, the craving for salt increases as forage DM decreases. In Florida, free-choice salt intake in the summer can be more than twofold the winter values. Salt craving increases into and throughout the rainy season (summer) and diminishes in the dry season (autumn/winter) [43]. This behavioral intake response is in contrast with the period of year that trace mineral nutrition would be considered most important. The typical calving season in Florida occurs in the winter and early spring, a time when cattle least crave salt and have the least voluntary intake of supplemental trace mineral. However, it is during this time that we would expect the greatest need for the physiological processes supported by trace minerals, such as colostrum formation, uterine involution, and rebreeding. There are other examples throughout the world where seasonal variation in salt craving impacts the effectiveness of voluntary intake of free-choice mineral supplements. For example, in some coastal regions of the world, salt-water intrusion will impact the Na content of forages and drinking water. In these regions, cattle may avoid salt-fortified supplements because of adequate or excessive Na intake form water and forage. Fortunately, if we understand these regional and climatic impacts, there are effective management interventions that may be adopted to protect against trace mineral deficiency resulting from inadequate free-choice intake.

The success of the free-choice, salt-based supplementation system is based on the concept that cattle are naturally seeking salt; therefore, it is essential that supplemental trace minerals be mixed directly in the salt carrier, not separate. Day-to-day variation in intake among individual animals should be expected. Cockwill et al. [44] evaluated individual intakes among grazing cow/calf pairs. They reported ranges of 0 to 974 g/d for cows and 0 to 181 g/d for calves. On average, cows visited the mineral 1.1 times/d, but over the 6-day evaluation period, only 22% of the calves recorded even a single visit to the feeder. In another study, Manzano et al. [45] reported a range in variation in free-choice mineral intake (reported as CV) of 77.5 to 108.4% among individual steers grazing cool season forages. These researchers also observed differences in the pattern of mineral feeder visits throughout the day, whereas in the autumn period steers concentrated their visits during the mid-day hours compared to a more uniform pattern of visits throughout the day in the spring period. The authors suggest that the steers targeted their visits to the mineral feeder during the daylight hours, fewer of which were available in the autumn period. The influence of time of day on visits to the mineral feeder was also reported by Ranches et al. [46]. In that study, Brahman cows visited the mineral feeder more often during the hottest hours of the day (13:00 to 20:59 h) compared to Black Angus cows. These breed-related differences are likely the result of the subtropical region of the country where this study was conducted. According to Braghieri et al. [47], cows are more engaged in grazing during morning and afternoon, which could affect visits to the mineral feeder. Brahman cattle are more tolerant to heat when compared to Angus cattle [48], thus presenting a breed x environment impact on free-choice mineral consumption among grazing beef cattle. The variation in visits to a mineral feeder, either influenced by forage type, season, or breed, have management implications, and should be considered when developing a mineral supplementation program.

A recent study focused on variation in free-choice mineral intake was conducted at North Dakota State University [49]. The study utilized the SmartFeed system (C-Lock, Inc., Rapid City, SD, USA) to monitor individual mineral supplement intake, number of visits to the feeder and duration of each individual visit. The results of the study show that cows and calves visit the mineral feeder together and calf mineral intake was positively correlated to the duration of time the cow spent at the mineral feeder. When separating cows into low intake (<90 g/d) and high intake (>90 g/d) groups, these authors reported greater liver mineral concentrations of Se and Cu among cows that consumed the greatest amount of free-choice mineral. These findings further support the importance of managing free-choice, salt-based mineral supplement intake to optimize cow mineral status. They also provide new evidence that calf learning behavior related to mineral feeder visiting is influenced by the cow, thus potentially impacting their current and future behavior for free-choice supplement intake and ultimately mineral status.

When attempting to determine the optimal salt inclusion level of a free-choice, trace mineral-fortified supplement, the average intake of the herd over a 1- to 2-week period should be the target. The level of salt inclusion can then be used as a tool to encourage or discourage mineral intake, as increased salt inclusion will reduce visits to the mineral feeder and overall supplement intake [46]. When assessing average herd intake, grazing cattle should not be offered straight salt separate or additional to the trace mineral fortified salt. This will complicate intake of the fortified salt supplement leading to the potential for trace mineral deficiency. This concept is best described by the failure of “cafeteria style” mineral supplementation systems to satisfy grazing cattle trace mineral requirements [50,51]. These systems are incorrectly designed around a false supposition that grazing cattle have a “nutritional wisdom” to consume different sources of supplemental minerals at a rate that will satisfy their individual requirement. Indeed, although there is ample evidence that ruminants possess an ability to learn to avoid consuming toxic plants [52], there is a lack of evidence supporting their ability to seek trace minerals during times of insufficiency.

Free-choice, salt-based supplements are available in both loose mixtures and compressed salt blocks. These different physical forms will impact supplement consumption. Mineral blocks (i.e., salt blocks) offer management conveniences related to labor because they often require less frequent replacement due to reduced intake by cattle. Block intake can be as much as 10% less than the consumption of supplements in loose form, potentially creating a gap between mineral consumption and cattle requirements [1]. On the other hand, the use of low moisture molasses blocks is a great technology for the delivery of minerals. Moriel et al. [53] evaluated the use of trace mineral fortified molasses blocks vs. loose mineral supplement with yearling calves and found that calves assigned to loose mineral supplement had lower total dry matter intake than calves assigned to molasses block fortified with trace minerals. Although there was a difference in total dry matter intake due to treatment, there were no differences on trace mineral status of calves. Similarly, Aubel et al. [54] reported lower intake for cows consuming loose mineral supplement vs. mineral fortified molasses blocks. Bailey et al. [55] evaluated the consumption of molasses blocks fortified with trace minerals vs. loose mineral supplements offered to mature cows. They reported greater consumption of blocks when compared to loose mineral supplement, which reflected in a greater intake of specific trace minerals such as Cu and Zn. In that study, the authors also reported a greater number of visits to the location where molasses blocks where placed when compared to the location where loose mineral supplement were located. These greater intakes observed for molasses blocks fortified with minerals are likely due to improved palatability of such supplements when compared to salt-based loose mineral supplements. Supplement palatability is an important factor when developing supplementation strategies, as it can be a valuable tool to manipulate cattle intake as well as cattle distribution within a pasture.

### 3.2. Fortification of Energy/Protein Supplements

The best method of insuring intake of supplemental trace minerals is through the fortification of energy and protein supplements. Most grazing beef cattle are offered supplemental energy and protein at certain times of the year when forage quantity or quality may be insufficient to supply these major nutrients. This is an excellent opportunity to utilize this delivery vehicle mechanism to supplement trace minerals to grazing cattle. Although variation in voluntary intake among cattle that are provided with energy and protein supplements is expected, it is less than the variation observed with loose, mineral mix supplements [56]. Many commercially formulated energy and protein supplements offer mineral fortification. Careful examination of the nutrient profile, matched to targeted intake, can determine if additional trace mineral supplementation is needed. If properly formulated, free-choice, salt-based supplementation could be replaced by energy and protein supplements during specific seasons of the year. In addition to formulation, management is paramount to success. Attention to animal dominance, adequate bunk space, feeding methods, and targeted intakes are essential [57]. When properly managed, this supplementation strategy can significantly impact the overall economics of nutrient supplementation of the grazing cowherd.

### 3.3. Trace Mineral Injections

Injectable sources of trace minerals have been available for several decades, particularly for Cu and Se applications. In the example of Cu, they have not been favorably adopted due to the propensity to create injection site reactions [58], which were sometimes severe depending on the preparation [59]. More recently, injectable multielement trace mineral products containing a combination of EDTA-bound Cu, Zn, and Mn with Na selenite have become more commonly used in grazing cattle. Among these products, MultiMin (MultiMin North America, Fort Collins, CO, USA) has been the subject for many published studies. Since the early 2010s, there have been a number of studies completed aimed at evaluating MultiMin on measures of performance and health of beef cattle. Pogge et al. [60] evaluated the trace mineral status of beef steers over a 15-day period following injection of MultiMin and reported elevated liver concentration of Cu and Se, at least through d 15. In a following study, this team reported increased liver Cu and Se concentrations through at least 30 d following injection [61]. In terms of immune competence, Arthington and Havenga [62] reported increased humoral immune responses in steers receiving MultiMin concurrent to a commercially available multivalent vaccine. This early finding has been expanded in later studies which illustrate the positive role that injectable trace minerals (MultiMin 90) exert in both humoral and cell mediated responses, when included in viral vaccination protocols [63,64,65]. This enhancement in immune function has resulted in improved morbidity outcomes when highly stressed beef calves are treated with injectable trace minerals (MultiMin 90) [66].

Injectable trace minerals have also been shown to improve reproductive performance among grazing beef cattle. Mundell et al. [67] provided two injections of trace minerals to beef cows grazing native grass prairie. The injections were administered approximately 100 d prior to calving and again at 30 d prior to artificial insemination. Under this treatment scenario, cows administered injectable trace minerals had a greater pregnancy outcome to artificial insemination. In contrast, Stokes et al. [68] reported no effect of repeated administrations of injectable trace minerals on pregnancy outcome to artificial insemination among developing beef heifers. In both studies, the protocol allowed free-choice access to trace mineral-fortified salt-based supplements. As described above, variation in individual animal intake is expected, which likely confounds the effects of the injectable trace mineral treatment. Nonetheless, trace mineral status at the time of breeding would likely impact the influence of additional trace mineral supply through injection on subsequent reproductive outcomes. The control heifers enrolled in the latter study [68] were confirmed to be mineral adequate by liver samples collected at breeding. In contrast, Mundell et al. [67] reported a large variation in mineral status of cows enrolled in their study using plasma trace mineral concentrations as an indicator. Although plasma trace mineral concentrations are a poorer estimate of status compared to liver tissue, these authors suggest that a portion of their cows may have been trace mineral inadequate despite the provision of free-choice trace mineral-fortified supplement.

Indeed, it is not surprising to conclude that trace mineral status of the cow or heifer at the time of injectable trace mineral administration would impact subsequent performance responses, such as reproduction. In a large study involving 799 heifers [69], injectable trace minerals were administered (or not) at the start of a 14-day timed-AI protocol. Free-choice, trace mineral-fortified supplement was provided to both treatment groups. Liver samples collected from a subset of heifers revealed adequate trace mineral status for both treatment groups. As a result, trace mineral injection had no impact on reproductive performance. The findings of these and other studies present an important consideration when evaluating the potential influence of trace mineral injection on the reproductive performance of grazing beef cattle. Current mineral status and the provision of additional sources of supplemental trace minerals are clearly important contributors to the likelihood of a positive outcome.

The influence of injectable trace minerals on male fertility has also been evaluated. In a large study conducted in Kansas [70], the effect of injectable trace minerals on the development of breeding soundness was evaluated using 488 young growing bulls of approximately 7 months of age. In their study, injection of trace minerals (or saline; control) was provided on d 0 when the bulls were approximately 7 months of age and again 90 d later at first breeding soundness examination. In their study, sperm motility and morphology were improved among bulls receiving injectable trace minerals. Although, no differences were noted for the total percentage of bulls passing the breeding soundness exam by 12 months of age, more bulls that failed the exam at 9 months of age (time of second injection) passed at 12 months of age if they received injectable trace minerals, compared to control bulls receiving saline. Initial plasma trace mineral status of bulls was assessed at the start of the study. Although plasma trace mineral concentrations are of limited value, as noted by the authors, they did report marginal plasma Zn concentrations using references ranges suggested by Puls [71]. Zinc is known to be an important trace mineral for development and support of normal spermatogenesis and the source and level of dietary Zn has been previously shown to influence attainment of breeding soundness in developing beef bulls [30].

Although the use of injectable trace minerals is a convenient method to address the mineral needs of beef cattle, this technology should not replace traditional mineral supplementation programs. Instead, injectable trace minerals should be used as nutritional tool to improve mineral status of cattle prior to challenging events such as calving, weaning, and breeding. Additionally, injectable trace minerals may serve as a complement to traditional oral supplementation strategies, particularly in production systems having trouble managing routine delivery and/or intake of free-choice supplements. Examples may include extensive rangeland systems, seasonal grazing of mountain meadows, and seasonally flooded pastures. In these systems, injectable trace mineral delivery would be limited to the timing of other management practices which involve gathering of cattle, such as branding, breeding, and weaning.

### 3.4. Biofortification

Biofortification of forages is a noteworthy advancement in field of trace mineral supplementation of grazing cattle, particularly for Se. This methodology is based on the application of trace minerals as fertilizers to plants or amendments to soil. In the case of Se, biofortification of agronomic crops in Finland has been responsible for improving the Se status of the livestock and human population [72]. Research with forage crops has shown substantial increases in forage Se concentrations in Se-biofortified warm season grasses [73,74], cool-season grasses [75] and legume forages [76]. Selenium from Se-biofortified forage appears to be more available than inorganic Se at similar levels of intake when consumption is limited (<3 mg/d) [73]. Additionally, short-term access to Se-biofortified forages improves Se stores that may support cattle through subsequent periods of Se inadequacy [77]. Additional studies imply that Se-biofortified forages may improve the health and performance of beef cattle consuming these forages [78,79]. Among the areas of opportunity for improving the trace mineral nutrition of grazing cattle, biofortification hold tremendous opportunity for exploration and subsequent application.

### 3.5. Boluses and Drenches

Similar to trace mineral injections, trace mineral boluses and drenches are considered direct methods of trace mineral supplementation. In current production practices, their utilization is minimal, as there is paucity of data regarding the effectiveness of this strategy.

In the 1990s, there was an emergence of interest in the use of ruminal boluses containing Cu oxide needles. The concept was based on the bolus capsule dissolving in the rumen and thus releasing the Cu oxide needles to dissolve over time. Although this Cu supplementation strategy was effective to increase Cu status of the animal, it lost appeal following a series of studies revealing reduced BW gain and weaning weight of calves [80] and less in vivo digestibility of forage NDF and CP [81].

Long-acting trace mineral boluses containing Cu, Se, and Co have been developed more recently. The long-acting trace mineral boluses, which can last up to six months, are advantageous for cattle on expansive rugged topography areas, where the utilization of free-choice mineral supplements might be limited. In a long-term study (4 years) conducted in Arizona, Sprinkle et al. [82] reported that strategic supplementation using long-acting trace mineral bolus for cows grazing extensive rangeland, was successful in decreasing calving interval and increasing weaning weight of calves born to cows supplemented with mineral bolus, which provided 156, 5.9, and 3.4 mg daily of Cu, Co, and Se, respectively. In another study, Jackson et al. [83] evaluated several single-use, pulse-dose trace mineral products (injectable, drench, paste, and bolus), and reported that injectable trace minerals were effective at quickly increasing trace mineral concentrations in plasma and liver, while long-acting boluses may be of value if a gradual increase in liver trace mineral concentrations is desired. Approximately 120 d were required before boluses began to increase liver concentrations of certain trace minerals. Inversely, the use of oral products, such as drenches and paste, evaluated in this same study, had no effect on plasma or liver trace mineral concentrations of trace mineral-adequate cattle.

## 4. Feed Ingredient Sources of Supplemental Trace Minerals

### 4.1. Inorganic Trace Minerals

Inorganic trace minerals (carbonates, chlorides, sulfates, oxides, etc.) are recognized by the molecule arrangement with oxygen, chloride or other noncarbon-based compounds. Inorganic minerals have been used in livestock diets and supplements for many years and are still frequently used in the formulation of mineral supplements due to their availability and cost [2]. Although inorganic trace minerals are often used in ruminant supplements, these mineral sources often have lesser biological availability and stability when compared to other mineral sources. Among inorganic trace minerals, sulfate and chloride forms are generally the most bioavailable, followed by carbonates, with oxides being the least bioavailable. Copper and Fe oxides should not be included in ruminant diets and/or supplements due to their low bioavailability. Although price per unit of metal delivered varies among all trace mineral sources, inorganic trace minerals are typically the least expensive options for supplement formulations.

### 4.2. Organic Trace Minerals

Beginning in the mid-1990s, there was an increasing amount of interest in improving the trace mineral nutrition of livestock through improved bioavailability of new sources of trace minerals, namely organic trace minerals. Over a 25+ year period many different sources of organic trace minerals were introduced into the feed ingredient marketplace. When referring to trace mineral source, the term “organic” is generic and encompasses numerous examples of trace elements (predominantly divalent Cu, Zn, and Mn) covalently bound to an organic ligand. The American Association of Feed Control Officials [84] define organic trace minerals as seven different complexes; including metal (specific amino acid) complexes, metal amino acid complexes, metal amino acid chelates, metal proteinates, metal polysaccharide complexes, metal propionates, and yeast derivative complexes. Each of these complexes differ from another and often create confusion when describing the studies published using organic trace minerals. Gayathri and Panda [85] provides a review of each of these categories and different studies assessing bioavailability of organic trace minerals.

Although most Animal Scientists agree that organic trace minerals offer increased bioavailability in nonruminant species, this advantage is less clear and certainly more complicated in ruminants [86]. Research investigating the impacts of organic trace minerals on grazing production systems has been highly variable. This is likely due to the impacts that the rumen microbial population and fiber fractions in forage-based diets [87] exert on mineral x mineral interactions (e.g., formation of mineral complexes). Although organic trace minerals might be expected to avoid some of those interactions, the rumen most certainly complicates the process beyond that which would be anticipated in a monogastric system.

Marques et al. [88] reported positive responses in calves born to cows supplemented with organic trace minerals during late gestation. In their study, calves had 11% greater BW at weaning when born from cows receiving organic sources (amino acid complex) of Cu, Mn, Zn, and Co compared to cows receiving no supplemental mineral. Conversely, Ahola et al. [89] reported reduced calf weaning weight, expressed as kg of calf weaned per cow exposed, when cowherds were provided supplemental Cu, Zn, and Mn from both organic (mineral proteinates) and inorganic sources (sulfates). However, reproductive performance was improved among cows receiving supplemental Cu, Zn, and Mn vs. no supplemental sources of the same elements with a trend for improved AI pregnancy rate among cows receiving organic vs. inorganic sources of these elements. In contrast, Ahola et al. [90] reported a tendency for lesser overall pregnancy rate (60-day breeding season) over a 2-year study investigating beef cows receiving organic (mineral proteinate) or inorganic sources (sulfates) of Cu, Zn, and Mn. It is likely that age and production status may be impactful to production responses observed when organic trace mineral supplementation strategies are applied to grazing cow/calf production systems. Arthington and Swenson [43] reported on a 3-year study investigating the impact of supplemental Cu, Zn, Mn, and Co from organic (amino acid complex) and inorganic sources (CuSO_4_, ZnO_4_, MnO, and CoCO_3_). In that study, reproductive performance was not impacted by mineral source in mature cows; however, cows in their first and second parity (3 and 4 y of age) had greater pregnancy outcome and shorter calving interval when supplemented with organic vs. inorganic sources of Cu, Zn, Mn, and Co.

The impact of organic minerals on measures of beef calf health and post-weaning performance has also been investigated. Marques et al. [88] reported improved post-weaning morbidity among calves born to cows receiving organic vs. inorganic sources of Cu, Zn, Mn, and Co during late gestation. In another study investigating a similar source of organic trace minerals, Kegley et al. [91] reported improved growth performance with a tendency for reduced treatment for illness among weaned, shipping-stressed beef calves receiving supplemental organic (amino acid complex) vs. inorganic sources (sulfates) of Cu, Zn, Mn, and Co. However, in a later study [92], these authors reported no impact of Cu, Zn, and Mn source (inorganic, organic, or hydroxychloride) on growth performance and measures of health and morbidity among shipping-stressed beef calves. Variability among these and other studies could be attributed to many factors, such as, differences in the initial health status of the cattle, basal diet composition, breed and other external influences.

Semen quality also appears to be impacted by organic trace mineral supplementation. Rowe et al. [93] reported improved sperm motility among mature bulls receiving organic (amino acid complex) vs. inorganic sources (sulfates) of Cu, Zn, Mn, and Co. These authors suggest that sperm motility is the most important semen quality parameter and thus, organic trace mineral supplementation could enhance bull fertility. This response is likely the result of Zn, which is known to be important for the maintenance and integrity of physiological processes involving rapidly dividing cells, such as spermatogenesis. In an earlier study [30], Arthington et al. reported improved breeding soundness exam passage rate and greater percentage of normal sperm cells among developing Angus bulls receiving organic (metal proteinate) vs. inorganic (sulfate) sources of supplemental Zn.

Organic sources of Se differ from the organic Cu, Zn, and Mn studies described here. As a dietary ingredient, organic Se is generally offered as a form of selenomethionine or Se yeast, which predominantly contains Se in the form of selenomethionine [86]. In studies comparing inorganic Se (Na selenate or Na selenite) to organic Se, results typically favor larger increases in Se accumulation into milk and soft tissue among cattle consuming the organic Se sources. This response is likely a competitive amino acids response resulting in the nonspecific incorporation of selenomethionine vs. methionine [94].

### 4.3. Hydroxychloride Trace Minerals

More recently, a newer source of inorganic trace minerals has become available to the feed industry. Hydroxychloride sources of Cu, Zn, and Mn have been evaluated in several ruminant production models over the past 10+ years. These mineral sources have low water solubility [95,96] and in forage-fed ruminants, hydroxychloride sources of Cu and Zn are less soluble in the rumen and bind less tightly with solid digestion than sulfate sources of the same metals [97]. These ruminal characteristics may explain, at least partially, the alleged increase in Cu bioavilability of hydroxychloride vs. sulfate sources [96]. In a study conducted over two consecutive years [98], reproductive performance and calf weaning weight did not differ among cows and calves receiving Cu, Zn, and Mn from hydroxychloride vs. a blend of organic and inorganic sources, despite increased liver tissue accumulation of Cu and Zn among the hydroxychloride-supplemented cows.

Cattle have evolved an acute sense of taste which likely resulted in the bovine’s adaptation toward taste sensory as the primary nutrient sensing mechanism. This sensory mechanism is well developed with over twice the number of taste buds compared to humans [99]. In a previous study, pre-weaned calves were shown to have a taste aversion to mineral-concentrated creep feed [100]. Although not specifically evaluated in that study, the aversion was suspected to be the result of a “metallic-taste” sensitivity [101] in calves consuming the mineral-fortified supplement containing soluble sources (sulfate and organic) of Cu, Zn, and Mn. This theory was further examined in a later study [102] where beef calves were shown to preferentially consume creep feed supplements fortified with hydroxychloride vs. sulfate or organic sources of Cu, Zn, and Mn. Further studies support this preferential intake behavior in other supplementation strategies common for grazing beef cattle, such as cooked molasses tubs [103] and free-choice, salt-based supplements [104]. In the latter study [104], trace mineral solubility was assessed in a rainfall simulation model. Those findings revealed increased losses of Cu, Zn and Mn metal when salt-based supplements are formulated with sulfate or organic sources vs. hydroxychloride sources and exposed to rainfall simulation.

## 5. Trace Mineral Antagonists

The trace mineral nutrition of grazing cattle can be impacted by several factors including the presence of mineral antagonists in grazed forage and in energy and protein supplements. Mineral antagonistic interactions can take place in the digestive tract and at the site of metabolism [33]. These interactions can cause trace mineral deficiencies, which can be grouped into two broad categories; primary and secondary deficiency. Primary mineral deficiencies are the result of the consumption of feeds that are naturally low in one or more trace minerals, resulting in delayed observable signs of deficiency. Secondary mineral deficiencies are typically more common and are the consequence of the consumption of one or more mineral antagonists that interfere with the normal metabolism of another mineral [105]. Several antagonistic mineral interactions are known to result in changes in mineral retention and metabolic function in the body; however, considering the trace minerals focused on in this review, mineral antagonists of main interest are Fe, Mo, and S.

### 5.1. Iron Antagonism

Iron is an essential element for cattle (50 mg/kg) [6] and it is the second most common trace metal in the earth’s surface and therefore is found in virtually all sources of cattle feed. A considerable amount of Fe is also consumed by grazing cattle from soil intake. With great availability around the world, Fe deficiency is rarely observed in cattle [29]. In fact, Fe is more likely to be a concern due to its ability to antagonize other trace minerals, most notably Cu, Mn, and Zn.

According to Mills [106] the first adverse effects of a high Fe intake on Cu metabolism were observed with the development of hypocupremia in cattle consuming irrigation waters rich in Fe. Bremner et al. [107] reported differences in response to Fe inclusion in cattle diets to be dependent on rumen development. In their study, pre-ruminant calves receiving milk supplemented with 500 mg of Fe/kg of DM, did not show any negative effects on Cu status, specifically liver Cu retention. However, when calves were fed a barley grain and barley straw diet, the addition of 250 mg of Fe/kg of DM resulted in reduced liver and plasma Cu concentrations. Interestingly, Mullis et al. [108] reported breed differences on Cu status of steers fed high dietary Fe. Steers were assigned to one of four diets containing different sources of Cu and Zn (5 mg Cu and 25 mg supplemental Zn/kg DM) and were supplemented with 1000 mg Fe/kg DM (as FeSO_4_). Simmental steers had less serum ceruloplasmin, and serum and liver Cu concentrations throughout the study, when compared to Angus steers, suggesting a greater Cu requirement. Additionally, neither Cu sources were cable of preventing declines on serum Cu and ceruloplasmin concentrations, regardless of breed.

A potential explanation for the effects of Fe on Cu is related to the disassociation of ferrous sulfide complexes in the low pH of the abomasum. In this scenario, sulfide may be able to react with Cu, forming insoluble Cu-sulfide complexes [109]. Currently, the maximum tolerable concentration of Fe for cattle is established at 500 ppm [6].

### 5.2. Molybdenum Antagonism

**Figure 1:**
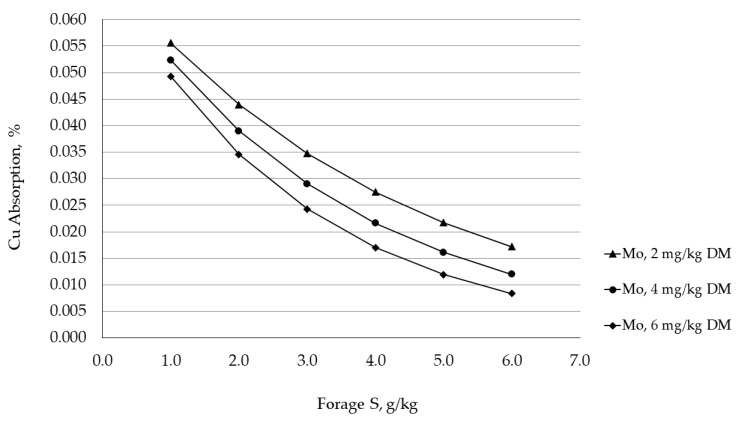
Hypothetical impact of the Mo × S interaction on Cu absorption in cattle.

Molybdenum is an essential trace element required by all animals; however, cattle requirements for Mo are not defined. Nonetheless, the current maximum tolerable concentration of Mo for cattle is 5 ppm. Although reports of Mo deficiency are very rare [6], the antagonistic impact of Mo on Cu metabolism has been recognized for many years and is exacerbated by S. This interaction occurs through the creation of ruminal thiomolybdates formed when molybdate reacts with sulfide, produced by rumen microorganisms via the reduction of sulfate. Consequently, thiomolybdates associate with solid rumen digesta (bacteria, protozoa and undigested feed particles) forming insoluble complexes with Cu, therefore reducing Cu absorption and potentially leading to deficiency [20,86]. Equations have been developed to aid in the determining the impact of Mo and S on Cu status of cattle [110]. Although these tools are helpful, the complex mineral-to-mineral interactions in ruminants preclude nutritionists from fully understanding these relationships. Using equations derived from Agricultural Research Council [111], Gordon Carstens (Texas A&M University) prepared a hypothetical dataset which illustrates the impact of dietary Mo and S on the efficiency of Cu absorption (Figure 1).

Ward and Spears [112] evaluated the long-term effects of low Cu diets with or without supplemental Mo (5 mg/kg of DM) on Cu status of growing steers. In their study, supplemental Mo decreased plasma Cu, ceruloplasmin concentration, and superoxide dismutase activity during the growing and finishing phases for steers not receiving supplemental Cu. However, Mo supplementation did not affect these parameters in Cu-supplemented steers, suggesting that Cu supplementation can reverse the negative effects observed with high-Mo diets. Molybdenum is an essential trace element required by all animals; however, cattle requirements for Mo are not defined. Nonetheless, the current maximum tolerable concentration of Mo for cattle is 5 ppm. Although reports of Mo deficiency are very rare [6], the antagonistic impact of Mo on Cu metabolism has been recognized for many years and is exacerbated by S. This interaction occurs through the creation of ruminal thiomolybdates formed when molybdate reacts with sulfide, produced by rumen microorganisms via the reduction of sulfate. Consequently, thiomolybdates associate with solid rumen digesta (bacteria, protozoa and undigested feed particles) forming insoluble complexes with Cu, therefore reducing Cu absorption and potentially leading to deficiency [21,87].

Recent findings from Thorndyke et al. [113] reported on the impacts of Mo consumed through water sources, which is a problematic situation for grazing cattle in some parts of the world. In their study, Mo consumed in water impacted Cu absorption and retention to a lesser extent than Mo supplemented in the diet. Steers were assigned to one of three treatments; no Mo supplementation (control); or 5.0 mg Mo/kg DM from sodium molybdate dihydrate in the diet; or 1.5 mg Mo/L from sodium molybdate dihydrate delivered in the drinking water. Molybdenum intake was greater for Mo supplemented steers, when compared to steers assigned to control treatment, regardless of supplementation method. Nonetheless, the apparent absorption and retention of Cu in steers supplemented with Mo in the water did not differ from steers assigned to control treatment. However, Mo-supplemented steers had similar apparent absorption and retention of Cu, regardless of supplementation method. The authors attributed these findings to a possible rumen bypass mechanism when Mo was supplemented in the water, as a major portion of water consumed can enter the abomasum directly via the esophageal groove, therefore reducing the possible interaction with S in the rumen.

### 5.3. Sulfur Antagonism

Sulfur is an essential macro-mineral (approximately 0.15% of diet DM) [6]; however, requirements for S are not well defined, particularly for grazing cattle. The maximum tolerable concentration of S is currently set at 0.3–0.5% of diet DM [6]. Sulfur is found naturally in nearly all feedstuffs and can vary widely from inorganic salt to organic S-containing amino acids. The S intake of cattle is often impacted by the use of S-containing fertilizers [13]; high-S byproducts (i.e., distillers grains, sugarcane molasses) [14]; high-sulfate water sources; and atmospheric deposition of S in forages (i.e., acid rain). A functional rumen tends to serve as a reducing environment for most elements, therefore, sulfate and even elemental S (i.e., flours of sulfur) can be reduced to sulfide in the rumen. It is important, therefore, to consider all sources of S consumed, regardless of chemical form, and estimate total S intake. If concentrations exceed 0.3%, which is approximately 30 g/d for a mature beef cow, Cu and Se status is likely to decrease over time despite adequate supplementation of these elements. When estimating total S intake, water sources may be an important contributor. However, a water’s odor is not a good indication of the S content. Often people will characterize water with a “sulfur smell” as having a high-S concentration. The odor is derived from hydrogen sulfide volatilizing off the surface of the water. Humans can detect the smell of hydrogen sulfide in the parts per billion, which is likely too low to contribute substantially to the S level in the total diet. Water tests should be conducted that provide a total S analysis. Coupled with an estimate of intake, S from water should be included into forage and supplement S contributions to derive an overall total.

As briefly explained previously, S impacts Cu metabolism in association with Mo, through the formation of thiomolybdates in the rumen. Nonetheless, S can impact Cu and Se metabolism directly through the formation of insoluble sulfide complexes [33]. Additionally, S and Se have similar physical and chemical properties, resulting in decreased bioavailability of Se when cattle consume diets with a high S concentration [86].

Arthington et al. [14] investigated the Cu status of grazing heifers that were supplemented with molasses supplements (approximately 0.78% S), a commonly used supplement with naturally high S concentration. In that study, heifers (approximately 350 kg of BW) were randomly assigned to one of three treatments, which provided 100 mg of supplemental Cu daily as inorganic Cu, organic Cu, or no Cu supplementation (control), corresponding to approximately 10 ppm assuming a DMI of 2.5% of BW. Throughout the study, non-supplemented heifers had the least liver and ceruloplasmin concentrations when compared to heifers supplemented with Cu. These findings are likely due to a combined effect of a lack of supplemental Cu and the antagonism of S naturally present in molasses. In another study, Arthington [114] investigated the impact of molasses supplementation on measures of Se status of steers, which were fed either a molasses or a corn-based diet, while supplemented with sodium selenite (3 mg/head daily). After a 90-day supplementation period, steers provided corn-based supplements had greater liver Se concentrations than steers consuming molasses-based supplements suggesting that that dietary S, derived from sugarcane molasses, may antagonize liver tissue accumulation of Se in cattle.

Although the negative impacts of S on Cu and Se metabolism of cattle are widely accepted, different breeds of cattle respond differently to the antagonism. Ranches et al. [115] demonstrated that the Cu and Se response to high S diets is influenced by cattle breed. In their study, Angus and Brahman cows were subjected to a high S diet (50 g of supplemental S/cow daily) devoid of supplemental Cu and Se. This supplementation strategy was later followed by removal of S from diet and supplementation of Cu and Se (100 and 3 mg/d of Cu and Se, respectively). Several Cu and Se status markers were evaluated throughout the study, demonstrating that Brahman compared with Angus cows, were more efficient at maintaining adequate Cu status when consuming a Cu-inadequate diet with high S. In contrast, Angus cows compared with Brahman cows, were more efficient at maintaining adequate Se status when consuming a Se-inadequate diet with high S.

## 6. Conclusions

The trace mineral nutrition of grazing beef cattle is essential and is often a complicated component of the management program. Through the annual cycle, forage is the primary source of trace mineral supply to the grazing animal, which can be impacted by several factors, such as soil characteristics, forage species, season, and climate. Trace mineral deficiencies, most notably Se, Cu, Zn, Mn, Co, and I, can be observed when cattle rely solely on forages to meet the requirements. Furthermore, the presence of trace mineral antagonists such as Fe, Mo, and S, which are commonly found in grazed forage, can complicate the success of trace mineral supplementation program. Nonetheless, several trace mineral supplementation strategies are available, such as fortification of energy and protein supplements, biofortification, injectable trace minerals, and boluses. Regardless of options, trace mineral-fortified, salt-based, free-choice supplements are the most common strategy used. These supplements can be formulated to meet the cattle requirements using different trace mineral sources, which are grouped into different source categories, such as, inorganic trace minerals, organic trace minerals, and hydroxychloride trace minerals. Irrespective of the choice of supplementation method, trace mineral nutrition is essential for proper development, performance, health, and reproduction of cattle. Efforts to meet the trace mineral requirements of grazing beef cattle are vital for the overall productivity of the cattle grazing system.

## Figures and Tables

**Table 1:** Changes in trace mineral requirements of beef cattle from 1984 to 2016 ^1^.

Mineral, mg/kg	6th NRC, 1984 [7]	7th NRC, 1996–2000 [8]	8th NRC, 2016 [6]	Maximum Tolerable Concentration ^2^
Chromium	-	-	-	1000
Cobalt	0.10	0.10	0.15	25
Copper	8	10	10	40
Iodine	0.50	0.50	0.50	50
Iron	50	50	50	500
Manganese ^3^	40	40	40	1000
Molybdenum	-	-	-	5
Nickel	-	-	-	50
Selenium	0.20	0.10	0.10	5
Zinc	30	30	30	500

^1^ The trace mineral requirements were summarized for mature cows (gestating and early lactation). ^2^ The maximum tolerable concentrations were according to the latest beef cattle requirement publication [6]. ^3^ Manganese requirement for growing and finishing cattle was set at 20 mg/kg in the 7th NRC edition [8] remaining equal in the latest NRC edition [6].

## Data Availability

Not applicable.
